# A Framework for Constructing, Estimating, and Validating the Bivariate Generalized Log-Gamma Distribution

**DOI:** 10.1016/j.mex.2026.104231

**Published:** 2026-10-23

**Authors:** Arief Rachman Hakim, Purhadi, Jerry Dwi Trijoyo Purnomo

**Affiliations:** a Department of Statistics, Faculty of Science and Data Analytics, Institut Teknologi Sepuluh Nopember, Surabaya, 60111, Indonesia; b Department of Statistics, Faculty of Science and Mathematics, Universitas Diponegoro, Semarang, 50275, Indonesia

**Keywords:** Bivariate generalized log-gamma distribution, Maximum likelihood estimation, BHHH algorithm, Diagnostic plot

## Abstract

The generalized log-gamma (GLG) distribution provides a flexible framework for modelling continuous data with different directions and degrees of skewness. However, its univariate form cannot represent dependence between two related response variables. This method article develops a bivariate generalized log-gamma (BGLG) distribution from the three-parameter GLG model. The proposed procedure includes construction of the joint probability density function, derivation of its statistical properties, formulation of the likelihood function, parameter estimation, simulation, and distributional adequacy assessment. The validity of the proposed density is established by showing that its integral over the bivariate sample space equals one. Because the maximum likelihood score equations have no closed-form solutions, the parameters are estimated iteratively using the Berndt–Hall–Hall–Hausman algorithm. The adequacy of the BGLG distribution is assessed using a chi-square quantile plot. An illustrative simulation using 500 observations under a single parameter setting produced asymmetric marginal distributions and positive dependence, while the chi-square diagnostic closely followed the reference line, with minor upper-tail deviations, providing initial support for the proposed BGLG construction.

Our approach involves:

• Constructing the BGLG joint density and deriving its properties.
• Estimating parameters by maximum likelihood using the BHHH algorithm.
• Evaluating the distribution through simulation and chi square diagnostics.

## Graphical abstract

**Figure:**
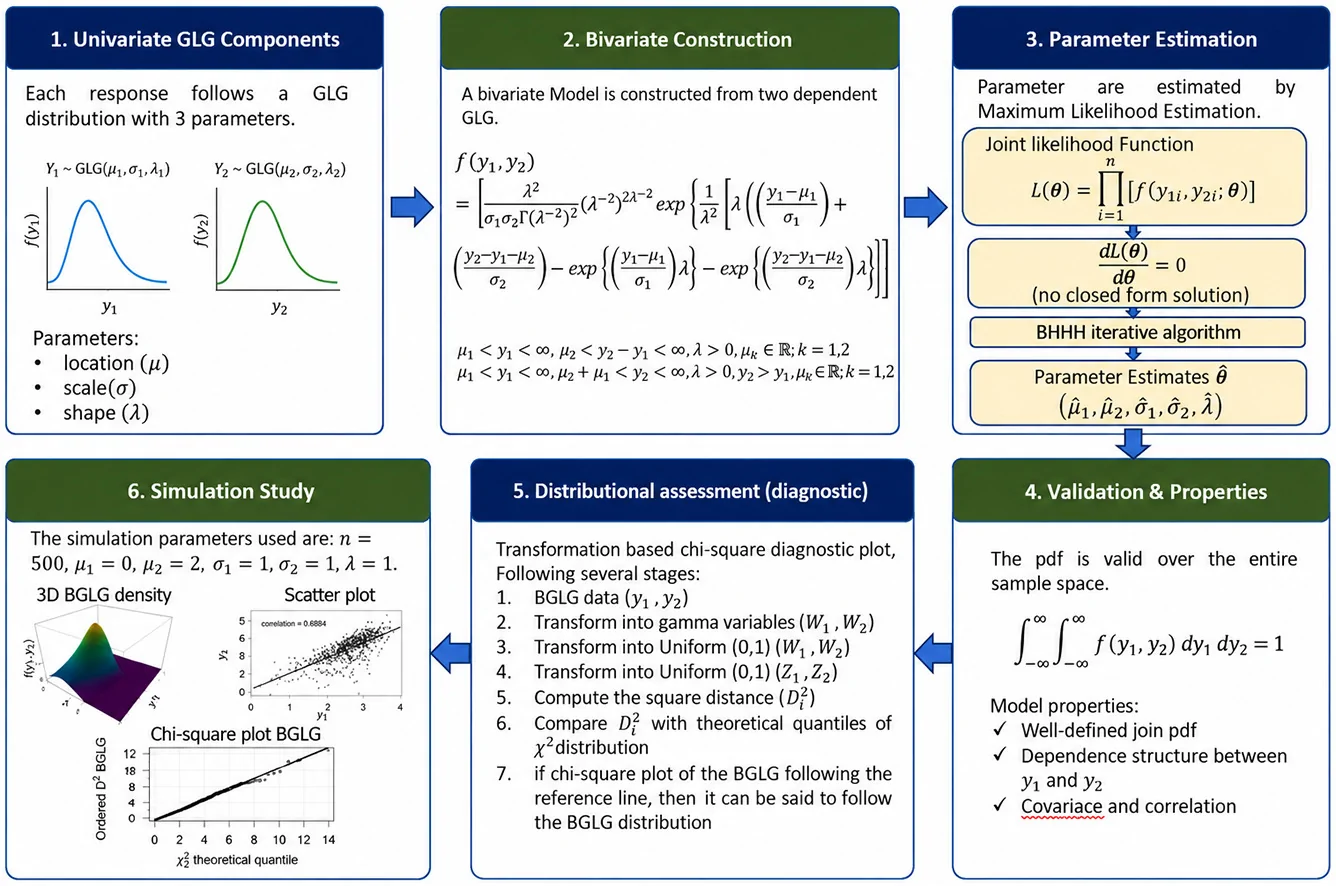

## Specifications table

*This table provides general information on your method*.

**Table:** 

**Subject area**	Mathematics and Statistics
**More specific subject area**	Probability distributions; bivariate statistical modeling; maximum likelihood estimation; numerical optimization; distributional adequacy assessment
**Name of your method**	BGLG construction, estimation, and diagnostic assessment method
**Name and reference of original method**	The method extends the univariate generalized log-gamma distribution of Prentice [2,10] and employs the BHHH numerical algorithm of Hall et al. [8]
**Resource availability**	The complete R code used for data generation, simulation, parameter estimation, and diagnostic assessment is provided as Supplementary Material.

## Background

The Generalized Log-Gamma (GLG) distribution is an extension of the single-parameter gamma distribution, which is equivalent to the single-parameter gamma log distribution [1]. The GLG distribution has two additional parameters, namely the location parameter (μ) and the scale parameter (σ), which extends the flexibility of the gamma distribution of a single parameter. Unlike the ordinary gamma distribution, which is restricted to positive observations, the GLG distribution can accommodate both negatively and positively skewed data through three parameters: location (μ), scale (σ), and shape (λ). The Generalized Log-Gamma distribution form was introduced by Prentice (1974) [2] and also introduced the form of modelling and parameter estimation. Then the development of the Generalized Log-Gamma distribution was carried out by Young (1987) [3] and Ortega et. al (2009) [4] includes distribution models and parameter estimation. The main advantage of GLG distributions lies in the flexibility that is able to accommodate skewness distributions to the left and right and the random variables in the GLG distribution are members of the real number set while gamma distributions require positive random variables.

Despite these advantages, the standard GLG distribution is univariate and therefore cannot directly describe the dependence between two related continuous responses. This limitation motivates the development of a bivariate generalized log-gamma (BGLG) distribution. In the proposed procedure, two dependent GLG components are combined through a variable transformation, and the corresponding Jacobian is used to derive the joint probability density function. The validity of the resulting density is established by showing that its integral over the entire bivariate sample space is equal to one.

The BGLG parameters are estimated using maximum likelihood estimation. Because the resulting score equations do not have closed-form solutions, numerical optimization is required. Therefore, a numerical method is needed to obtain the value of the parameter estimator. There are several numerical methods that can be used to solve non-closed form equations, including the Minorization Maximization (MM) algorithm [5], Broyden-Fletcher-Goldfarb-Shanno (BFGS) [6] and Newton Raphson [7] and Berndt-Hall-Hall-Hausman (BHHH) [8,9]. Among the available iterative procedures, the Berndt–Hall–Hall–Hausman (BHHH) algorithm is employed because it updates the parameter vector using the outer product of individual score contributions and is directly compatible with likelihood-based estimation. The proposed framework also includes a simulation procedure and a transformation-based chi-square diagnostic plot for assessing whether paired continuous observations are adequately represented by the fitted BGLG distribution. Together, these components provide a reproducible procedure for constructing, estimating, simulating, and evaluating the BGLG model.

## Method details

Univariate Generalized Log-Gamma Distribution was first studied by Prentice in 1974 [2] and Lawless in 1980 [1]. The GLG distribution has two additional parameters, namely the location parameter (μ) and the shape parameter (λ), which extends the flexibility of the gamma distribution of a single parameter. The univariate generalized log gamma form with 3 parameters, namely the location (μ), scale(σ), and shape (λ) parameters. Suppose Y is a random variable that follows the GLG distribution with the parameter μ,σ,λ. So the probability density function (pdf) of Y is shown in equation 1 as follows [10,11]:

(1) $$f\left(y|\mu ,\sigma ,\lambda \right)\;=\frac{\left|\lambda \right|}{\sigma \Gamma \left(\lambda^{-2}\right)}{\left(\lambda^{-2}\right)}^{\lambda^{-2}}exp\left\{\frac{1}{\;\lambda^{2}}\left[\left(\frac{y-\mu }{\sigma }\right)\lambda -\;exp\left\{\left(\frac{y-\mu }{\sigma }\right)\lambda \right\}\right]\right\}$$

with y,μ,λ∈R,λ≠0,σ>0.

Where μ,σ,λ successively are the parameters of location, scale, shape and $\Gamma \left(\lambda^{-2}\right)=\int_{0}^{\infty }t^{\lambda^{-2}-1}\;e^{-t}dt$ are gamma functions, if Y∼GLG(μ,σ,λ) then obtained:

1. $E\left(Y\right)=\;\mu +\sigma \;\left[\frac{\psi \left(\lambda^{-2}\right)-log\left(\lambda^{-2}\right)}{\left|\lambda \right|}\right]$

with $\psi \left(\lambda^{-2}\right)={\left[log(\Gamma (\lambda^{-2}))\right]}^{\prime }$ is a function of digamma. According to Abramowitz & Stegun (1964) [12], the digamma function for *x* is define $\psi \left(x\right)=-\gamma -\frac{1}{x}+\sum_{n=1}^{\infty }\left(\frac{x}{n(x+n)}\right)$ with γ is the Euler-Mascheroni constant (≈ 0.5772156649).

2. $Var\left(Y\right)=\;\sigma^{2}\;\frac{\psi^{\prime }\left(\lambda^{-2}\right)}{\lambda^{2}}$

with $\psi^{\prime }\left(\lambda^{-2}\right)={\left[log(\Gamma (\lambda^{-2}))\right]}^{{}^{\prime \prime }}-\psi^{2}\left(\lambda^{-2}\right)$ is a trigamma fuction.

3. F$\left(y\right)=I\left[\lambda^{-2},\lambda^{-2}exp\left(\lambda \frac{y-\mu }{\sigma }\right)\right]\;$is a Comulative Density Function.

with a incomplete gamma function

$$I\left[\lambda^{-2},\lambda^{-2}exp\left(\lambda \frac{y-\mu }{\sigma }\right)\right]=\frac{1}{\Gamma \left(\lambda^{-2}\right)}\int_{0}^{\lambda^{-2}exp\left(\lambda \frac{y-\mu }{\sigma }\right)}z^{\lambda^{-2}-1}e^{-z}\;dz$$

Some special distributions can be formed from the first GLG distribution if λ=0, the density function of the Normal distribution (0.1) is obtained.

$$f\left(y|0,1,\lambda =k\right)=\frac{1}{\sqrt{2\pi }}exp\left(-\frac{1}{2}y^{2}\right);k\to \infty$$

Second, if λ=1, then the density function is obtained from the *Extreme Value Type* 1 distribution (μ,σ).

$$f\left(y|\mu ,\sigma ,\lambda =1\right)=\;\frac{1}{\sigma }exp\left(-\frac{1}{\sigma }\left(y-\mu \right)-exp\left(\frac{1}{\sigma }\left(y-\mu \right)\right)\right)$$

with y∈R,μ∈R,σ∈R.

One of the characteristics of the GLG distribution is that if λ < 0 then the pdf of Y tends to have a slope to the right, then if λ > 0 then the pdf of Y then tends to have a slope to the left. Fig. 1 illustrates a pdf of the GLG distribution with parameter changes. Figure (A) a plot with the parameter λ < 0, σ = 6 with various values μ. Figure (B) a plot with the parameter λ = 0, σ = 6 with various values μ. Figure (C) of a plot with parameters λ > 0, σ = 6 with various value μ.

**Fig. 1:**
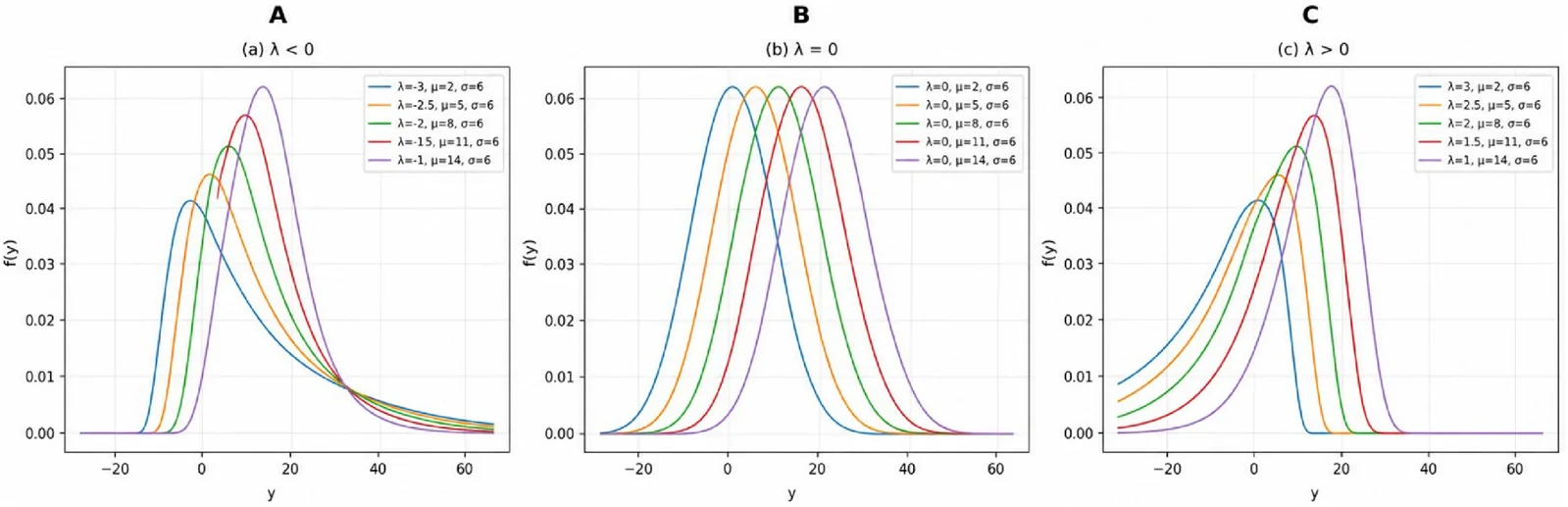
Probability density functions of the GLG distribution under different location and shape parameters.

The BGLG distribution was developed from the univariate form of GLG, this new form allows for a deeper and broader analysis. This is because the BGLG form of distribution is based on the concept of bivariate, which allows the use of two variables in the distribution model. This concept also allows the development of new methods of analysis with the concept of bivariate regression. The concept of BGLG is the main gap in this research when compared to previous researches. General procedure for developing the BGLG distribution in this study are as follows:

1. Define the bivariate transformation based on a univariate model dan construct the joint BGLG probability density function
2. Determine the *likelihood* and *ln-likelihood* functions based on equation 2
3. Make the first derivative of the *ln-likelihood* function for each of the estimated parameters and then equalize zero.
4. Estimate the parameters using the BHHH algorithm
5. Evaluate the proposed distribution (BGLG distribution testing)

Step 1: Define the bivariate transformation based on a univariate model dan construct the joint BGLG probability density function.

Suppose $Y_{1}\;$and $Y_{2}\;$are two non-independent random variables that follow the GLG distribution with the parameter (μ,σ,λ),then a Bivariate Generalized Log Gamma distribution can be formed with a pdf of $Y_{1}\;$and $Y_{2}$ shown in equation (2). Suppose $Z_{1},Z_{2}$ is a random variable that is free of each other (independent) with $Z_{k}∼GLG\left(\mu_{k},\;\sigma_{k},\lambda \right);k=1,2.$

$$Y_{1}=Z_{1}$$

$$Y_{2}=Z_{1}+Z_{2}$$

where the space of $\left(Z_{1},Z_{2}\right)$ is $\Omega_{\left(z_{1},z_{2}\right)}=\left\{\left(z_{1},z_{2}\right)|\;\mu_{k}<z_{k}<\infty ;\;\mu_{k}\in R,k=1,2\right\}$and the space of $\left(Y_{1},Y_{2}\right)$ is $\Omega_{\left(Y_{1},Y_{2}\right)}=\left\{\left(y_{1},y_{2}\right)|\;\mu_{k}<y_{k}-y_{k-1}<\infty ;\mu_{k}\in R,k=1,2\right\}$

$$Y_{1}=Z_{1}\to \;\mu_{1}<y_{1}<\infty$$

$$Y_{2}=Z_{1}+Z_{2}\to \;\mu_{1}+\;\mu_{2}<y_{2}<\infty$$

$$Y_{2}=Z_{1}+Z_{2}\to \;\mu_{2}<y_{2}-y_{1}<\infty$$

a function $(Y_{1},Y_{2})\;$is a function that maps $\Omega_{\left(Z_{1},Z_{2}\right)}\;$to $\Omega_{\left(Y_{1},Y_{2}\right)}$ until it is obtained:

$$Z_{1}=\omega_{1}\left(Y_{1},Y_{2}\right)=Y_{1}$$

$$Z_{2}=\omega_{2}\left(Y_{1},Y_{2}\right)=Y_{2}-Y_{1}$$

The Jacobian matrix of the equation is:

$$J=\left[\begin{array}{cc} \frac{\partial z_{1}}{\partial y_{1}} & \frac{\partial z_{1}}{\partial y_{2}}\\ \frac{\partial z_{2}}{\partial y_{1}} & \frac{\partial z_{2}}{\partial y_{2}} \end{array}\right]=\left[\begin{array}{cc} 1 & 0\\ -1 & 1 \end{array}\right]$$

The determinants of the Jacobian matrix are:

det(J)=|J|=1.1−(−1.0)=1

So that the PDF is shared from a random variable $Z_{1},Z_{2}$, assuming $\lambda_{1}\;$= $\lambda_{2}=\lambda$ is shown in the following equation 2:

$$h\left(z_{1},z_{2}\right)=h\left(z_{1}\right)h\left(z_{2}\right)$$

$$h\left(z_{1},z_{2}\right)=\left[\frac{\left|\lambda \right|}{\sigma \Gamma \left(\lambda^{-2}\right)}{\left(\lambda^{-2}\right)}^{\lambda^{-2}}exp\left\{\frac{1}{\;\lambda^{2}}\left[\left(\frac{z_{1}-\mu_{1}}{\sigma_{1}}\right)\lambda -\;exp\left\{\left(\frac{z_{1}-\mu_{1}}{\sigma_{1}}\right)\lambda \right\}\right]\right\}\right]\times$$

$$\left[\frac{\left|\lambda \right|}{\sigma \Gamma \left(\lambda^{-2}\right)}{\left(\lambda^{-2}\right)}^{\lambda^{-2}}exp\left\{\frac{1}{\;\lambda^{2}}\left[\left(\frac{z_{2}-\mu_{2}}{\sigma_{2}}\right)\lambda -\;exp\left\{\left(\frac{z_{2}-\mu_{2}}{\sigma_{2}}\right)\lambda \right\}\right]\right\}\right]$$

then

$$f\left(y_{1},y_{2}\right)=h\left(z_{1},z_{2}\right)\;\left|J\right|$$

$$f\left(y_{1},y_{2}\right)=h\left(y_{1}\right)h\left(y_{2}-y_{1}\right)\;\times \;1$$

$$=\left[\frac{\left|\lambda \right|}{\sigma_{1}\Gamma \left(\lambda^{-2}\right)}{\left(\lambda^{-2}\right)}^{\lambda^{-2}}exp\left\{\frac{1}{\;\lambda^{2}}\left[\left(\frac{y_{1}-\mu_{1}}{\sigma_{1}}\right)\lambda -\;exp\left\{\left(\frac{y_{1}-\mu_{1}}{\sigma_{1}}\right)\lambda \right\}\right]\right\}\right]\times$$

$$\left[\frac{\left|\lambda \right|}{\sigma_{2}\Gamma \left(\lambda^{-2}\right)}{\left(\lambda^{-2}\right)}^{\lambda^{-2}}exp\left\{\frac{1}{\;\lambda^{2}}\left[\left(\frac{y_{2}-y_{1}-\mu_{2}}{\sigma_{2}}\right)\lambda -\;exp\left\{\left(\frac{y_{2}-y_{1}-\mu_{2}}{\sigma_{2}}\right)\lambda \right\}\right]\right\}\right]$$

(2) $$\begin{array}{c} f\left(y_{1},y_{2}\right)\\ =\left[\frac{\lambda^{2}}{\sigma_{1}\sigma_{2}{\left(\Gamma \left(\lambda^{-2}\right)\right)}^{2}}{\left(\lambda^{-2}\right)}^{2\lambda^{-2}}exp\left\{\frac{1}{\;\lambda^{2}}\left[\lambda \left(\left(\frac{y_{1}-\mu_{1}}{\sigma_{1}}\right)+\left(\frac{y_{2}-y_{1}-\mu_{2}}{\sigma_{2}}\right)\right)-\;exp\left\{\left(\frac{y_{1}-\mu_{1}}{\sigma_{1}}\right)\lambda \right\}-\;exp\left\{\left(\frac{y_{2}-y_{1}-\mu_{2}}{\sigma_{2}}\right)\lambda \right\}\right]\right\}\right] \end{array}$$

where:

$$\mu_{1}<y_{1}<\infty ,\mu_{2}<y_{2}-y_{1}\langle \infty ,\lambda \rangle 0,\mu_{k}\in R;k=1,2$$

$\mu_{1}<y_{1}<\infty ,\mu_{2}+\mu_{1}\langle y_{2}\langle \infty ,\lambda \rangle 0,y_{2}\rangle y_{1},\mu_{k}\in R;k=1,2$ and $f(y_{1},y_{2})=0$ for others.

Some of the properties of the BGLG distribution are as follows:

$$E\left[Z_{1}\right]=\mu_{1}+\sigma_{1}\;\left[\frac{\psi \left(\lambda^{-2}\right)-log\left(\lambda^{-2}\right)}{\left|\lambda \right|}\right]$$

$$E\left[Z_{2}\right]=\mu_{2}+\sigma_{2}\;\left[\frac{\psi \left(\lambda^{-2}\right)-log\left(\lambda^{-2}\right)}{\left|\lambda \right|}\right]$$

$$E\left[Y_{1}\right]=E\left[Z_{1}\right]=\mu_{1}+\sigma_{1}\;\left[\frac{\psi \left(\lambda^{-2}\right)-log\left(\lambda^{-2}\right)}{\left|\lambda \right|}\right]$$

$$E\left[Y_{2}\right]=E\left[Z_{1}+Z_{2}\right]=E\left[Z_{1}\right]+E\left[Z_{2}\right]$$

$$=\left(\mu_{1}+\sigma_{1}\;\left[\frac{\psi \left(\lambda^{-2}\right)-log\left(\lambda^{-2}\right)}{\left|\lambda \right|}\right]\right)+\left(\mu_{2}+\sigma_{2}\;\left[\frac{\psi \left(\lambda^{-2}\right)-log\left(\lambda^{-2}\right)}{\left|\lambda \right|}\right]\right)$$

$$=\mu_{1}+\mu_{2}+\sigma_{1}\;\left[\frac{\psi \left(\lambda^{-2}\right)-log\left(\lambda^{-2}\right)}{\left|\lambda \right|}\right]+\sigma_{2}\;\left[\frac{\psi \left(\lambda^{-2}\right)-log\left(\lambda^{-2}\right)}{\left|\lambda \right|}\right]$$

$$=\mu_{1}+\mu_{2}+\;\left[\frac{\psi \left(\lambda^{-2}\right)\left(\sigma_{1}+\sigma_{2}\right)-log\left(\lambda^{-2}\right)\left(\sigma_{1}+\sigma_{2}\right)}{\left|\lambda \right|}\right]$$

It is known that $\text{var}\left[Z_{1}\right]=\sigma_{1}^{2}\;\frac{\psi^{\prime }\left(\lambda^{-2}\right)}{\lambda^{2}}\;$and $\text{var}\left[Z_{2}\right]=\sigma_{2}^{2}\;\frac{\psi^{\prime }\left(\lambda^{-2}\right)}{\lambda^{2}}$, then:

$$Var\left[Y_{1}\right]=var\left[Z_{1}\right]=\sigma_{1}^{2}\;\frac{\psi^{\prime }\left(\lambda^{-2}\right)}{\lambda^{2}}$$

$$Var\left[Y_{2}\right]=var\left[Z_{1}+Z_{2}\right]=var\left[Z_{1}\right]+var\left[Z_{2}\right]$$

$$Var\left[Z_{2}\right]=\sigma_{1}^{2}\;\frac{\psi^{\prime }\left(\lambda^{-2}\right)}{\lambda^{2}}+\;\sigma_{2}^{2}\;\frac{\psi^{\prime }\left(\lambda^{-2}\right)}{\lambda^{2}}$$

$$Var\left[Z_{2}\right]=\left(\sigma_{1}^{2}+\sigma_{2}^{2}\right)\;\frac{\psi^{\prime }\left(\lambda^{-2}\right)}{\lambda^{2}}$$

$$E\left[Y_{1}Y_{2}\right]=E\left[Z_{1}\left(Z_{1}+Z_{2}\right)\right]=E\left[Z_{1}^{2}+Z_{1}Z_{2}\right]$$

$$=E\left[Z_{1}^{2}\right]+E\left[Z_{1}Z_{2}\right]$$

$$=var\left(Z_{1}\right)+{\left(E\left(Z_{1}\right)\right)}^{2}+E\left[Z_{1}\right]E\left[Z_{2}\right]$$

$$=var\left(Y_{1}\right)+\;E\left[Z_{1}\right]\left(E\left[Z_{2}\right]+E\left[Z_{1}\right]\right)$$

$$=var\left(Y_{1}\right)+\;E\left[Y_{1}\right]\left(E\left[Z_{2}\right]+E\left[Z_{1}\right]\right)$$

$$E\;=var\left[Y_{1}\right]+E\left[Y_{1}\right]E\left[Y_{2}\right]$$

$$=\sigma_{1}^{2}\;\frac{\psi^{\prime }\left(\lambda^{-2}\right)}{\lambda^{2}}+\left[\mu_{1}+\sigma_{1}\;\left[\frac{\psi \left(\lambda^{-2}\right)-log\left(\lambda^{-2}\right)}{\left|\lambda \right|}\right]\right]\left[\mu_{1}+\mu_{2}+\;\left[\frac{\psi \left(\lambda^{-2}\right)\left(\sigma_{1}+\sigma_{2}\right)-log\left(\lambda^{-2}\right)\left(\sigma_{1}+\sigma_{2}\right)}{\left|\lambda \right|}\right]\right]$$

covariance

$$cov\left[Y_{1},Y_{2}\right]=cov\;\left[Z_{1},Z_{1}+Z_{2}\right]=cov\;\left[Z_{1},Z_{1}\right]+cov\;\left[Z_{1},Z_{2}\right]$$

$$cov\;=var\;\left[Z_{1}\right]+0=var\;\left[Y_{1}\right]$$

$$co\;=\sigma_{1}^{2}\;\frac{\psi^{\prime }\left(\lambda^{-2}\right)}{\lambda^{2}}$$

correlation

$$\rho_{Y_{1},Y_{2}}=\frac{cov\left[Y_{1},Y_{2}\right]}{\sqrt{Var\left[Y_{1}\right]Var\left[Y_{2}\right]}}=\frac{var\;\left[Y_{1}\right]}{\sqrt{Var\left[Y_{1}\right]Var\left[Y_{2}\right]}}$$

Step 2: determine the likelihood and log-likelihood functions based on equation 2.The parameter estimation stage begins by creating a likelihood function based on equation 2 as follows:
  $$L\left(\mu_{1},\mu_{2},\sigma_{1},\sigma_{2},\lambda \right)$$
  $$=\prod_{i=1}^{n}\left[\frac{\lambda^{2}}{\sigma_{1}\sigma_{2}{\left(\Gamma \left(\lambda^{-2}\right)\right)}^{2}}{\left(\lambda^{-2}\right)}^{2\lambda^{-2}}\exp\left\{\frac{1}{\lambda^{2}}\left[\lambda \left(\frac{y_{1i}-\mu_{1}}{\sigma_{1}}+\frac{y_{2i}-y_{1i}-\mu_{2}}{\sigma_{2}}\right)-\exp\left(\lambda \frac{y_{1i}-\mu_{1}}{\sigma_{1}}\right)-\exp\left(\lambda \frac{y_{2i}-y_{1i}-\mu_{2}}{\sigma_{2}}\right)\right]\right\}\right]$$

Forming the log likelihood form of equation 2 is defined in equation 3 as follows:

$$l\left(\mu_{1},\mu_{2},\sigma_{1},\sigma_{2},\lambda \right)$$

(3) $$=n\ln\left(\frac{\lambda^{2}}{{\left(\Gamma \left(\lambda^{-2}\right)\right)}^{2}}\right)-n\ln\left(\sigma_{1}\right)-n\ln\left(\sigma_{2}\right)+2n\lambda^{-2}\ln\left(\lambda^{-2}\right)+\frac{1}{\lambda^{2}}\sum_{i=1}^{n}\left[\lambda \left(\frac{y_{1i}-\mu_{1}}{\sigma_{1}}+\frac{y_{2i}-y_{1i}-\mu_{2}}{\sigma_{2}}\right)-\exp\left(\lambda \frac{y_{1i}-\mu_{1}}{\sigma_{1}}\right)-\;\text{exp}\left(\lambda \frac{y_{2i}-y_{1i}-\mu_{2}}{\sigma_{2}}\right)\right]$$

Step 3: Derive the score equations for each model parameter and set them equal to zero.
  $$\frac{\partial l\left(\mu_{1},\mu_{2},\sigma_{1},\sigma_{2},\lambda \right)}{\partial \mu_{1}}=-\frac{1}{\lambda \sigma_{1}}\sum_{i=1}^{n}\left[1-e^{\lambda d_{1i}}\right]=0$$
  $$\frac{\partial l\left(\mu_{1},\mu_{2},\sigma_{1},\sigma_{2},\lambda \right)}{\partial \mu_{2}}=-\frac{1}{\lambda \sigma_{2}}\sum_{i=1}^{n}\left[1-e^{\lambda d_{2i}}\right]=0$$
  $$\frac{\partial l\left(\mu_{1},\mu_{2},\sigma_{1},\sigma_{2},\lambda \right)}{\partial \sigma_{1}}=-\frac{n}{\sigma_{1}}+\frac{1}{\lambda \sigma_{1}^{2}}\sum_{i=1}^{n}\left[\left(y_{1i}-\mu_{1}\right)\left(1-e^{\lambda d_{1i}}\right)\right]=0$$
  $$\frac{\partial l\left(\mu_{1},\mu_{2},\sigma_{1},\sigma_{2},\lambda \right)}{\partial \sigma_{2}}=-\frac{n}{\sigma_{2}}+\frac{1}{\lambda \sigma_{2}^{2}}\sum_{i=1}^{n}\left[\left(y_{2i}-y_{1i}-\mu_{2}\right)\left(1-e^{\lambda d_{2i}}\right)\right]=0$$
  $$\frac{\partial l\left(\mu_{1},\mu_{2},\sigma_{1},\sigma_{2},\lambda \right)}{\partial \lambda }=\frac{2n}{\lambda }+4n\lambda^{-6}\psi \left(\lambda^{-2}\right)-2n\lambda^{-3}\left[1+2\ln\left(\lambda^{-2}\right)\right]-\frac{2}{\lambda^{3}}\sum_{i=1}^{n}\left[\lambda d_{i}-e^{\lambda d_{1i}}-e^{\lambda d_{2i}}\right]$$
  $$+\frac{1}{\lambda^{2}}\sum_{i=1}^{n}\left[d_{i}-d_{1i}e^{\lambda d_{1i}}-d_{2i}e^{\lambda d_{2i}}\right]=0$$

with:

$$d_{1i}=\frac{y_{1i}-\mu_{1}}{\sigma_{1}};$$

$$d_{2i}=\frac{y_{2i}-y_{1i}-\mu_{2}}{\sigma_{2}};$$

$$d_{i}=d_{1i}+d_{2i}$$

Step 4: estimate the parameters using the BHHH algorithm

The score equations for the model parameters do not admit closed-form solutions and are therefore solved numerically using the Berndt–Hall–Hall–Hausman (BHHH) algorithm [8]. It is defined that the vector parameter is $\theta_{BGLG}={\left[\mu_{1},\mu_{2},\sigma_{1},\sigma_{2},\lambda \;\right]}^{T}$ and the parameter estimation vector is ${\hat{\theta }}_{BGLG}={\left[{\hat{\mu }}_{1},{\hat{\mu }}_{2},{\hat{\sigma }}_{1},{\hat{\sigma }}_{2},\hat{\lambda }\;\right]}^{T}$.

The steps to obtain the value of the BGLG parameter estimator ${\hat{\theta }}_{BGLG}={\left[{\hat{\mu }}_{1},{\hat{\mu }}_{2},{\hat{\sigma }}_{1},{\hat{\sigma }}_{2},\hat{\lambda }\;\right]}^{T}$using the BHHH iteration are shown in the following algorithm:

1. Let $\{(y_{1i},y_{2i});i=1,2,\ldots ,n\}\;$be a random sample from the Bivariate Generalized Log-Gamma distribution. The parameter vector is defined as $\theta_{BGLG}={\left[\mu_{1},\mu_{2},\sigma_{1},\sigma_{2},\lambda \;\right]}^{T}$. where $\mu_{1}$and $\mu_{2}$ are location parameters, $\sigma_{1}>0\;$and $\sigma_{2}>0$ are scale parameters, and λ is the common shape parameter. The log-likelihood function of BGLG refers to equation 3.
2. Determining the initial value of the model parameters ${\hat{\theta }}_{BGLG}^{\left(0\right)}={\left[{\hat{\mu }}_{1}^{\left(0\right)},{\hat{\mu }}_{2}^{\left(0\right)},{\hat{\sigma }}_{1}^{\left(0\right)},{\hat{\sigma }}_{2}^{\left(0\right)},{\hat{\lambda }}^{\left(0\right)}\;\right]}^{T}$, to ensure positive scale parameters, define $\eta_{1}=\text{log}\left(\sigma_{1}\right),\;\eta_{2}=\text{log}\left(\sigma_{2}\right),$ so that $\sigma_{1}=\exp\left(\eta_{1}\right),\;\sigma_{2}=\exp\left(\eta_{2}\right).$ The working parameter vector is $\phi_{BGLG}={\left(\mu_{1},\mu_{2},\eta_{1},\eta_{2},\lambda \right)}^{T}.$

Initial values may be determined from the marginal characteristics of the data:

$${\hat{\mu }}_{1}^{\left(0\right)}=\text{median}\left(Y_{1}\right),\;{\hat{\mu }}_{2}^{\left(0\right)}=\text{median}\left(Y_{2}\right),{\hat{\sigma }}_{1}^{\left(0\right)}=\text{sd}\left(Y_{1}\right),\;{\hat{\sigma }}_{2}^{\left(0\right)}=\text{sd}\left(Y_{2}\right),\;{\hat{\lambda }}^{\left(0\right)}=1.$$

The initial transformed scales are ${\hat{\eta }}_{1}^{\left(0\right)}=\log\left(\sigma_{1}^{\left(0\right)}\right),\;{\hat{\eta }}_{2}^{\left(0\right)}=\log\left(\sigma_{2}^{\left(0\right)}\right)$ and the working parameter estimation vector is ${\hat{\phi }}_{BGLG}^{\left(0\right)}={\left[{\hat{\mu }}_{1}^{\left(0\right)},{\hat{\mu }}_{2}^{\left(0\right)},{\hat{\eta }}_{1}^{\left(0\right)},{\hat{\eta }}_{2}^{\left(0\right)},{\hat{\lambda }}^{\left(0\right)}\;\right]}^{T}$

3. Forming Hessian approximation, for each observation pair $\left(y_{1i},y_{2i}\right)$, the individual score vector is
  $$g_{i}\left(\phi_{BGLG}\right)=\frac{\partial l_{i}\left(\phi_{BGLG}\right)}{\partial \phi_{BGLG}}=\left(\begin{array}{c} \frac{\partial l_{i}}{\partial \mu_{1}}\\ \frac{\partial l_{i}}{\partial \mu_{2}}\\ \frac{\partial l_{i}}{\partial \eta_{1}}\\ \frac{\partial l_{i}}{\partial \eta_{2}}\\ \frac{\partial l_{i}}{\partial \lambda } \end{array}\right)$$

The total score vector is

$$g\left(\phi_{BGLG}\right)=\sum_{i=1}^{n}g_{i}\left(\phi_{BGlG}\right)$$

$$H\left(g\left(\phi_{BGLG}\right)\right)=-\sum_{i=1}^{n}g_{i}\left(\phi_{BGLG}\right){\left[g_{i}\left(\phi_{BGLG}\right)\right]}^{T}$$

The condition that must be met to maximize $L(\phi_{BGLG})$ is that a Hessian matrix is required which is a negative definite matrix.

1. Specifies the tolerance limit (ε) for the convergence of ε > 0 and the maximum iteration limit of M.
in this study, the convergence tolerance was set to $\varepsilon =10^{-6}$ and the maximum number of iterations to M=1000, with the iteration terminated when the change in the working parameter vector between two successive iterations was less than $10^{-6}$ or when the maximum number of iterations was reached.
2. Starts the BHHH iteration of *m* = 0 with the following equation (4):
  (4) $${\hat{\phi }}_{BGLG}^{\left(m+1\right)}={\hat{\phi }}_{BGLG}^{\left(m\right)}-H^{-1}\left({\hat{\phi }}_{BGLG}^{\left(m\right)}\right)g\left({\hat{\phi }}_{BGLG}^{\left(m\right)}\right)$$
3. The iteration will stop if $∥{\hat{\phi }}_{BGLG}^{\left(m+1\right)}-{\hat{\phi }}_{BGLG}^{\left(m\right)}∥<\varepsilon$ or if the iteration has reached M iteration, then estimator of the BGLG model parameter will be obtained ${\hat{\phi }}_{BGLG}={\hat{\phi }}_{BGLG}^{\left(M\right)}$ or it can be write ${\hat{\phi }}_{BGLG}={\left[{\hat{\mu }}_{1},{\hat{\mu }}_{2},{\hat{\eta }}_{1},{\hat{\eta }}_{2},\hat{\lambda }\;\right]}^{T}$.
4. After convergence, the estimated working parameter vector is ${\hat{\phi }}_{BGLG}={\left[{\hat{\mu }}_{1},{\hat{\mu }}_{2},{\hat{\eta }}_{1},{\hat{\eta }}_{2},\hat{\lambda }\;\right]}^{T}\;$then scale parameters are transformed back to their original scale:
  $${\hat{\sigma }}_{1}=\text{exp}\left({\hat{\eta }}_{1}\right),\;{\hat{\sigma }}_{2}=\text{exp}\left({\hat{\eta }}_{2}\right)$$
5. Thus, the final BGLG parameter estimates are ${\hat{\theta }}_{BGLG}={\left[{\hat{\mu }}_{1},{\hat{\mu }}_{2},{\hat{\sigma }}_{1},{\hat{\sigma }}_{2},\hat{\lambda }\;\right]}^{T}$
6. Matrix obtained $\hat{Cov}\left({\hat{\theta }}_{BGLG}\right)=\hat{Cov}\left({\left[{\hat{\mu }}_{1},{\hat{\mu }}_{2},{\hat{\sigma }}_{1},{\hat{\sigma }}_{2},\hat{\lambda }\right]}^{T}\right)\underset{n\to \infty }{≅}-H^{-1}\left({\hat{\theta }}_{BGLG}\right)$, where is the main element of the diagonal matrix $\hat{Cov}\left({\hat{\theta }}_{BGLG}\right)$ is the value of $\hat{var}\left({\hat{\mu }}_{j}\right)$, $\hat{var}\left({\hat{\sigma }}_{j}\right)$ and $\hat{var}\left(\hat{\lambda }\right),$ with j=0,1,2,…,p
7. Then $se\left({\hat{\mu }}_{j}\right)=\sqrt{\hat{var}\left({\hat{\mu }}_{j}\right)\;}$, $se\left({\hat{\sigma }}_{j}\right)=\sqrt{\hat{var}\left({\hat{\sigma }}_{j}\right)\;}$ and $se\left(\;\hat{\lambda }\right)=\sqrt{\hat{var}\left(\hat{\lambda }\right)\;}$.
Step 5: Evaluate the proposed distribution (BGLG distribution testing)

The basic principle in detecting or identifying the 2 data variables is Bivariate Generalized Log-Gamma, which uses the same principle as the normal identification of multivariate[13,14]. However, although the principle is the same as the chi-square plot in normal multivariate, for Bivariate Generalized Log-Gamma (BGLG) the *Y_1_* and *Y_2_* data are first transformed into the standard form of BGLG, then to the Gamma distribution, then to the standard Normal distribution through the probability integral transform. The complete stages are as follows:

1. Suppose bivariate data $(y_{1i},y_{2i}),\;i=1,2,\ldots ,n$
2. The data is assumed to come from the BGLG distribution with the parameter $\theta_{BGLG}={\left[\mu_{1},\mu_{2},\sigma_{1},\sigma_{2},\lambda \;\right]}^{T}$
3. Because the population parameters are unknown, the parameters of the MLE estimation results of the BGLG distribution model are used, namely ${\hat{\theta }}_{BGLG}={\left[{\hat{\mu }}_{1},{\hat{\mu }}_{2},{\hat{\sigma }}_{1},{\hat{\sigma }}_{2},\hat{\lambda }\;\right]}^{T}$
4. Standardizing BGLG data, where the original data (Y_1_, Y_2_) is transformed into a standard form using:
  $${\overset{ˆ}{z}}_{1i}=\frac{y_{1i}-{\overset{ˆ}{\mu }}_{1}}{{\overset{ˆ}{\sigma }}_{1}}\;\text{and}\;{\overset{ˆ}{z}}_{2i}=\frac{y_{2i}-y_{1i}-{\overset{ˆ}{\mu }}_{2}}{{\overset{ˆ}{\sigma }}_{2}}$$
5. It further transforms the generalized variable log-gamma into a Gamma form. In generalized log-gamma distributions, the transformation $T=\lambda^{-2}\exp\left(\lambda Z\right)$
6. Therefore, for BGLG data used:
  $${\overset{ˆ}{T}}_{1i}={\overset{ˆ}{\lambda }}^{-2}\exp\left(\overset{ˆ}{\lambda }{\overset{ˆ}{z}}_{1i}\right)$$
  $${\overset{ˆ}{T}}_{2i}={\overset{ˆ}{\lambda }}^{-2}\exp\left(\overset{ˆ}{\lambda }{\overset{ˆ}{z}}_{2i}\right)$$
7. If the data follows the BGLG distribution, then:
  $${\overset{ˆ}{T}}_{1i},{\overset{ˆ}{T}}_{2i}\approx \text{Gamma}\left({\overset{ˆ}{\lambda }}^{-2},1\right)$$
8. Then by using the Gamma CDF transformation. CDF Gamma is used to convert a Gamma variable into a uniform variable. The transformations are:
  $${\overset{ˆ}{U}}_{1i}=F_{\Gamma }\left({\overset{ˆ}{T}}_{1i};{\overset{ˆ}{\lambda }}^{-2},1\right)$$
  $${\overset{ˆ}{U}}_{2i}=F_{\Gamma }\left({\overset{ˆ}{T}}_{2i};{\overset{ˆ}{\lambda }}^{-2},1\right)$$
9. Based on the probability integral transform, if *T* has a cumulative distribution function of F(T), then:
  U=F(T)∼U(0,1)
10. Thus, if the BGLG model is suitable, then:
  $${\overset{ˆ}{U}}_{1i},{\overset{ˆ}{U}}_{2i}\approx U\left(0,1\right)$$
11. The next step transforms the uniform variable to the standard normal using the standard normal CDF inverse:
  $${\overset{ˆ}{W}}_{1i}=\Phi^{-1}\left({\overset{ˆ}{U}}_{1i}\right)$$
  $${\overset{ˆ}{W}}_{2i}=\Phi^{-1}\left({\overset{ˆ}{U}}_{2i}\right)$$
12. If the model is appropriate, then the result of the transformation meets: ${\overset{ˆ}{W}}_{1i},{\overset{ˆ}{W}}_{2i}\approx N\left(0,1\right)$
13. Forming the BGLG diagnostic square spacing: ${\hat{D}}_{i}^{2}={\hat{W}}_{1i}^{2}+{\hat{W}}_{2i}^{2}$
14. Since the square sum of the two standard normal variables follows the chi-square distribution with two degrees of freedom, then: ${\hat{D}}_{i}^{2}\approx \chi_{2}^{2}$
15. Sorting the value of square spacing from smallest to largest ${\hat{D}}_{\left(1\right)}^{2}\leq {\hat{D}}_{\left(2\right)}^{2}\leq \cdots \leq {\hat{D}}_{\left(n\right)}^{2}$
16. Calculate the probability of plotting position for each sequence i:
  $$p_{i}=\frac{i-\frac{1}{2}}{n},\;i=1,2,\ldots ,n$$
17. Then the theoretical quantile of the chi-square distribution is calculated with two free degrees:
  $$q_{i}=F_{\chi_{2}^{2}}^{-1}\left(\frac{i-\frac{1}{2}}{n}\right)$$
18. create a chi-square plot BGLG with a pair of points:
  $$\left(q_{i},{\hat{D}}_{\left(i\right)}^{2}\right)$$

or in full: $\left(F_{\chi_{2}^{2}}^{-1}\left(\frac{i-\frac{1}{2}}{n}\right),{\hat{D}}_{\left(i\right)}^{2}\right)$

19. To the plot a reference line is added:
  y=x

If the plotted points lie close to the reference line y=x, the resulting squared diagnostic distances are graphically consistent with the theoretical $\chi_{2}^{2}$ quantiles, providing graphical support for the adequacy of the fitted BGLG distribution. Conversely, systematic deviations from the reference line may indicate a lack of distributional adequacy. This procedure should be interpreted as a graphical diagnostic rather than as a formal goodness-of-fit test.

## Method validation

The proposed BGLG method was validated through mathematical, graphical, and simulation-based assessments. First, the validity of the joint probability density function was established by showing that its integral over the entire bivariate sample space is equal to one[15]. The distributional shape was then examined using three-dimensional surface and contour plots to evaluate its asymmetry and dependence structure. A simulation involving 500 paired observations was conducted to assess whether the generated data retained the expected marginal characteristics and relationship between $Y_{1}$and $Y_{2}$. Finally, the adequacy of the fitted BGLG distribution was evaluated using a transformation-based chi-square diagnostic plot*.*

A pdf will be called a valid function as a probability density function if the total probability in the entire sample space is equal to 1.

$$\int_{-\infty }^{\infty }\int_{-\infty }^{\infty }f\left(y_{1},y_{2}\right)\;dy_{1}\;dy_{2}=1$$

with

$$\sigma_{1}>0,\;\sigma_{2}>0,\;\lambda \neq 0$$

$z_{1}=\frac{y_{1}-\mu_{1}}{\sigma_{1}};$ $\;z_{2}=\frac{y_{2}-y_{1}-\mu_{2}}{\sigma_{2}};$ $y_{1}=\mu_{1}+\sigma_{1}z_{1}$; $y_{2}=y_{1}+\mu_{2}+\sigma_{2}z_{2}$

Then equation 2 will change to equation 5 as follows:

(5) $$f\left(y_{1},y_{2}\right)=\frac{\lambda^{2}}{\sigma_{1}\sigma_{2}{\left\{\Gamma \left(\lambda^{-2}\right)\right\}}^{2}}{\left(\lambda^{-2}\right)}^{2\lambda^{-2}}\exp\left[\lambda^{-2}\left\{\lambda \left(z_{1}+z_{2}\right)-\exp\left(\lambda z_{1}\right)-\exp\left(\lambda z_{2}\right)\right\}\right]$$

So the transformation of $(z_{1},z_{2})\;$to $\left(y_{1},y_{2}\right)$ is

$$y_{1}=\mu_{1}+\sigma_{1}z_{1}$$

$$y_{2}=\mu_{1}+\mu_{2}+\sigma_{1}z_{1}+\sigma_{2}z_{2}$$

$$J=\left(\begin{array}{cc} \frac{\partial y_{1}}{\partial z_{1}} & \frac{\partial y_{1}}{\partial z_{2}}\\ \frac{\partial y_{2}}{\partial z_{1}} & \frac{\partial y_{2}}{\partial z_{2}} \end{array}\right)=\left(\begin{array}{cc} \sigma_{1} & 0\\ \sigma_{1} & \sigma_{2} \end{array}\right)$$

Then $\left|J\right|=\sigma_{1}\sigma_{2}-\left(0\right)\left(\sigma_{1}\right)=\sigma_{1}\sigma_{2}$

So that the differential element changes to

$$dy_{1}\;dy_{2}=\left|J\right|\;dz_{1}\;dz_{2}=\sigma_{1}\sigma_{2}\;dz_{1}\;dz_{2}$$

Suppose the total integral is

$$I=\int_{-\infty }^{\infty }\int_{-\infty }^{\infty }f\left(y_{1},y_{2}\right)\;dy_{1}\;dy_{2}$$

$$I=\int_{-\infty }^{\infty }\int_{-\infty }^{\infty }\frac{\lambda^{2}}{\sigma_{1}\sigma_{2}{\left\{\Gamma \left(\lambda^{-2}\right)\right\}}^{2}}{\left(\lambda^{-2}\right)}^{2\lambda^{-2}}\exp\left[\lambda^{-2}\left\{\lambda \left(z_{1}+z_{2}\right)-\exp\left(\lambda z_{1}\right)-\exp\left(\lambda z_{2}\right)\right\}\right]dy_{1}\;dy_{2}$$

with $dy_{1}\;dy_{2}=\sigma_{1}\sigma_{2}\;dz_{1}\;dz_{2}$

$$I=\int_{-\infty }^{\infty }\int_{-\infty }^{\infty }\frac{\lambda^{2}}{\sigma_{1}\sigma_{2}{\left\{\Gamma \left(\lambda^{-2}\right)\right\}}^{2}}{\left(\lambda^{-2}\right)}^{2\lambda^{-2}}\exp\left[\lambda^{-2}\left\{\lambda \left(z_{1}+z_{2}\right)-\exp\left(\lambda z_{1}\right)-\exp\left(\lambda z_{2}\right)\right\}\right]\sigma_{1}\sigma_{2}\;dz_{1}\;dz_{2}$$

$$I=\frac{\lambda^{2}}{{\left\{\Gamma \left(\lambda^{-2}\right)\right\}}^{2}}{\left(\lambda^{-2}\right)}^{2\lambda^{-2}}\int_{-\infty }^{\infty }\int_{-\infty }^{\infty }\text{exp}\left[\lambda^{-2}\left\{\lambda z_{1}-\exp\left(\lambda z_{1}\right)+\lambda z_{2}-\exp\left(\lambda z_{2}\right)\right\}\right]dz_{1}\;dz_{2}$$

$$I=\frac{\lambda^{2}}{{\left\{\Gamma \left(\lambda^{-2}\right)\right\}}^{2}}{\left(\lambda^{-2}\right)}^{2\lambda^{-2}}\int_{-\infty }^{\infty }\int_{-\infty }^{\infty }\text{exp}\left[\lambda^{-2}\left\{\lambda z_{1}-\exp\left(\lambda z_{1}\right)\right\}+\lambda^{-2}\left\{\lambda z_{2}-\exp\left(\lambda z_{2}\right)\right\}\right]dz_{1}\;dz_{2}$$

$$I=\frac{\lambda^{2}}{{\left\{\Gamma \left(\lambda^{-2}\right)\right\}}^{2}}{\left(\lambda^{-2}\right)}^{2\lambda^{-2}}\left[\int_{-\infty }^{\infty }\exp\left\{\lambda^{-2}\left(\lambda z_{1}-\exp\left(\lambda z_{1}\right)\right)\right\}dz_{1}\right]\times \left[\int_{-\infty }^{\infty }\exp\left\{\lambda^{-2}\left(\lambda z_{2}-\exp\left(\lambda z_{2}\right)\right)\right\}dz_{2}\right]$$

Since both integrals have the same shape, it is enough to calculate the following general integrals:

$$I_{z}=\int_{-\infty }^{\infty }\exp\left[\lambda^{-2}\left\{\lambda z-\exp\left(\lambda z\right)\right\}\right]dz$$

If $a=\lambda^{-2}$

$$I_{z}=\int_{-\infty }^{\infty }\exp\left[a\left\{\lambda z-\exp\left(\lambda z\right)\right\}\right]dz$$

Then use the transform

t=aexp(λz)

$$\lambda z=\log\left(\frac{t}{a}\right)$$

$$\frac{dt}{dz}=a\lambda \exp\left(\lambda z\right)$$

because aexp(λz)=t, then

$$\frac{dt}{dz}=\lambda t$$

dt=λtdz

$$dz=\frac{dt}{\lambda t}$$

To keep the integral positive when usedλ<0, an absolute value is:

$$dz=\frac{dt}{\left|\lambda \right|t}$$

The integral boundary changes to

z→−∞⇒t→0

z→∞⇒t→∞

$$I_{z}=\int_{0}^{\infty }\exp\left[a\left\{\log\left(\frac{t}{a}\right)-\frac{t}{a}\right\}\right]\frac{1}{\left|\lambda \right|t}\;dt$$

$$I_{z}=\int_{0}^{\infty }\exp\left[a\log\left(\frac{t}{a}\right)-t\right]\frac{1}{\left|\lambda \right|t}\;dt$$

$$I_{z}=\int_{0}^{\infty }\exp\left[a\log t-a\log a-t\right]\frac{1}{\left|\lambda \right|t}\;dt$$

$$I_{z}=\frac{1}{\left|\lambda \right|}\int_{0}^{\infty }t^{-1}\exp\left(a\log t-a\log a-t\right)\;dt$$

because

$$\exp\left(a\log t\right)=t^{a}$$

$$\exp\left(-a\log a\right)=a^{-a}\;\text{then}$$

$$I_{z}=\frac{1}{\left|\lambda \right|}\int_{0}^{\infty }t^{-1}t^{a}a^{-a}e^{-t}\;dt$$

$$I_{z}=\frac{1}{\left|\lambda \right|}a^{-a}\int_{0}^{\infty }t^{a-1}e^{-t}\;dt$$

Use the definition of gamma functions:

$$\Gamma \left(a\right)=\int_{0}^{\infty }t^{a-1}e^{-t}\;dt$$

then

$$I_{z}=\frac{1}{\left|\lambda \right|}a^{-a}\Gamma \left(a\right)=\frac{\Gamma \left(a\right)}{\left|\lambda \right|a^{a}}$$

Substitution back $=\lambda^{-2}$, obtained:

$$I_{z}=\frac{\Gamma \left(\lambda^{-2}\right)}{\left|\lambda \right|{\left(\lambda^{-2}\right)}^{\lambda^{-2}}}$$

Because it is integral $z_{1}\;$and $z_{2}$ has the same shape, then

$$I=\frac{\lambda^{2}}{{\left\{\Gamma \left(\lambda^{-2}\right)\right\}}^{2}}{\left(\lambda^{-2}\right)}^{2\lambda^{-2}}\left(I_{z}\right)\left(I_{z}\right)$$

$$I=\frac{\lambda^{2}}{{\left\{\Gamma \left(\lambda^{-2}\right)\right\}}^{2}}{\left(\lambda^{-2}\right)}^{2\lambda^{-2}}{\left(I_{z}\right)}^{2}$$

$$I=\frac{\lambda^{2}}{{\left\{\Gamma \left(\lambda^{-2}\right)\right\}}^{2}}{\left(\lambda^{-2}\right)}^{2\lambda^{-2}}{\left[\frac{\Gamma \left(\lambda^{-2}\right)}{\left|\lambda \right|{\left(\lambda^{-2}\right)}^{\lambda^{-2}}}\right]}^{2}$$

$$I=\frac{\lambda^{2}}{{\left\{\Gamma \left(\lambda^{-2}\right)\right\}}^{2}}{\left(\lambda^{-2}\right)}^{2\lambda^{-2}}\frac{{\left\{\Gamma \left(\lambda^{-2}\right)\right\}}^{2}}{\lambda^{2}{\left(\lambda^{-2}\right)}^{2\lambda^{-2}}}$$

I=1

Simplify the same factors:

• $\lambda^{2}\;$ Mutual Elimination
• ${\left\{\Gamma (\lambda^{-2})\right\}}^{2}$ Mutual Elimination
• ${\left(\lambda^{-2}\right)}^{2\lambda^{-2}}$ Mutual Elimination

Thus it is evident that

$$\int_{-\infty }^{\infty }\int_{-\infty }^{\infty }f\left(y_{1},y_{2}\right)\;dy_{1}\;dy_{2}=1$$

or simply

$$\int_{R^{2}}f\left(y_{1},y_{2}\right)\;dy_{1}\;dy_{2}=1$$

So the function $f(y_{1},y_{2})\;$is valid as the probability density function or PDF of the Bivariate Generalized Log-Gamma distribution, because the total probability is equal to one.

An illustrative simulation was conducted to demonstrate the behavior of the proposed BGLG distribution under a single parameter setting. The simulated data were generated according to the following model:

$$Y_{1}=\mu_{1}+\sigma_{1}Z_{1}\;\text{and}\;Y_{2}=Y_{1}+\mu_{2}+\sigma_{2}Z_{2}$$

with

$Z_{1},Z_{2}∼\text{Generalized}\;\text{Log}-\text{Gamma}\;\text{Standar}$.

The illustrative simulation parameters used n=500paired observations with,$\;\mu_{1}=0,\;\mu_{2}=2,\;\sigma_{1}=1,\sigma_{2}=1$ and a common shape parameter λ=1.

This single simulation configuration was used to illustrate the distributional characteristics of the proposed BGLG construction and should not be interpreted as a comprehensive assessment of its performance across different parameter settings..

Figs. 2 are the figures showing the probability density function (PDF) of the Bivariate Generalized Log-Gamma Regression distribution for two random variables, namely $Y_{1}\;and\;Y_{2}$ . In general, the drawing consists of two parts: a 3D surface plot at the top and a contour plot at the bottom. On the 3D plot, it can be seen that the distribution has one main peak. This peak indicates the area with the greatest probability density value, which is a combination of values $y_{1}$ and $y_{2}$ the most likely based on the parameters used. The higher the surface, the greater the value of $f(y_{1},y_{2})$. On the other hand, the horizontal and dark-colored part indicates an area with very little chance. The characteristic that appears of this distribution is that the shape is not perfectly symmetrical. The distribution surface appears to be slightly elongated and skewed, thus indicating a relationship or dependence between $Y_{1}$ and $Y_{2}$. When the value $Y_{1}$ increases, the center of density $Y_{2}$ also tends to shift upwards. This illustrates the pattern of positive relationships between the two response variables.

**Fig. 2:**
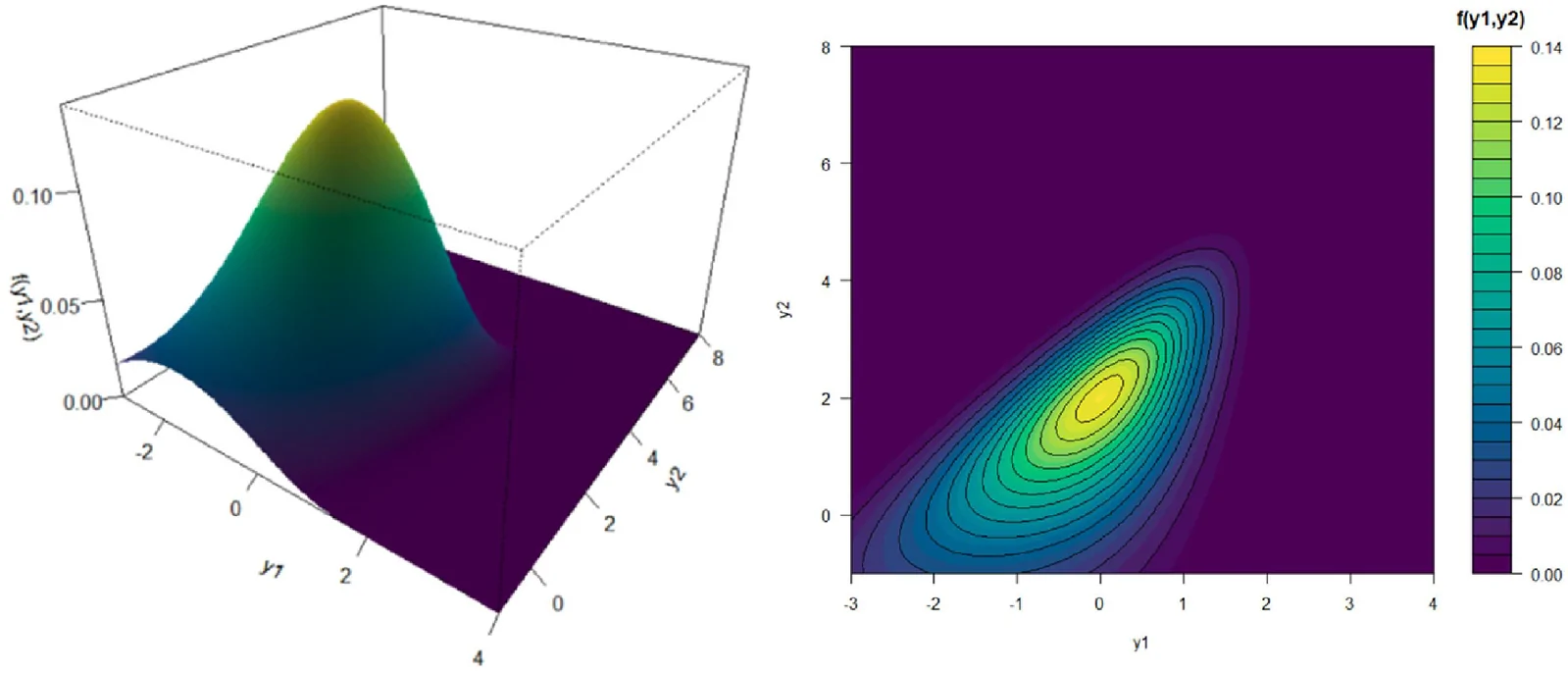
Three-dimensional surface and contour representations of the proposed BGLG density.

Interpretively, this figure shows that the Bivariate Generalized Log-Gamma distribution is able to describe bivariate data that are correlated with each other, have a certain density peak, and can accommodate tight or asymmetrical distribution patterns. This is one of the advantages of the generalized log-gamma distribution over the bivariate normal distribution, because this distribution is more flexible for data that have skewness and non-normal relationship patterns. Histogram images are generated as Fig. 3.

**Fig. 3:**
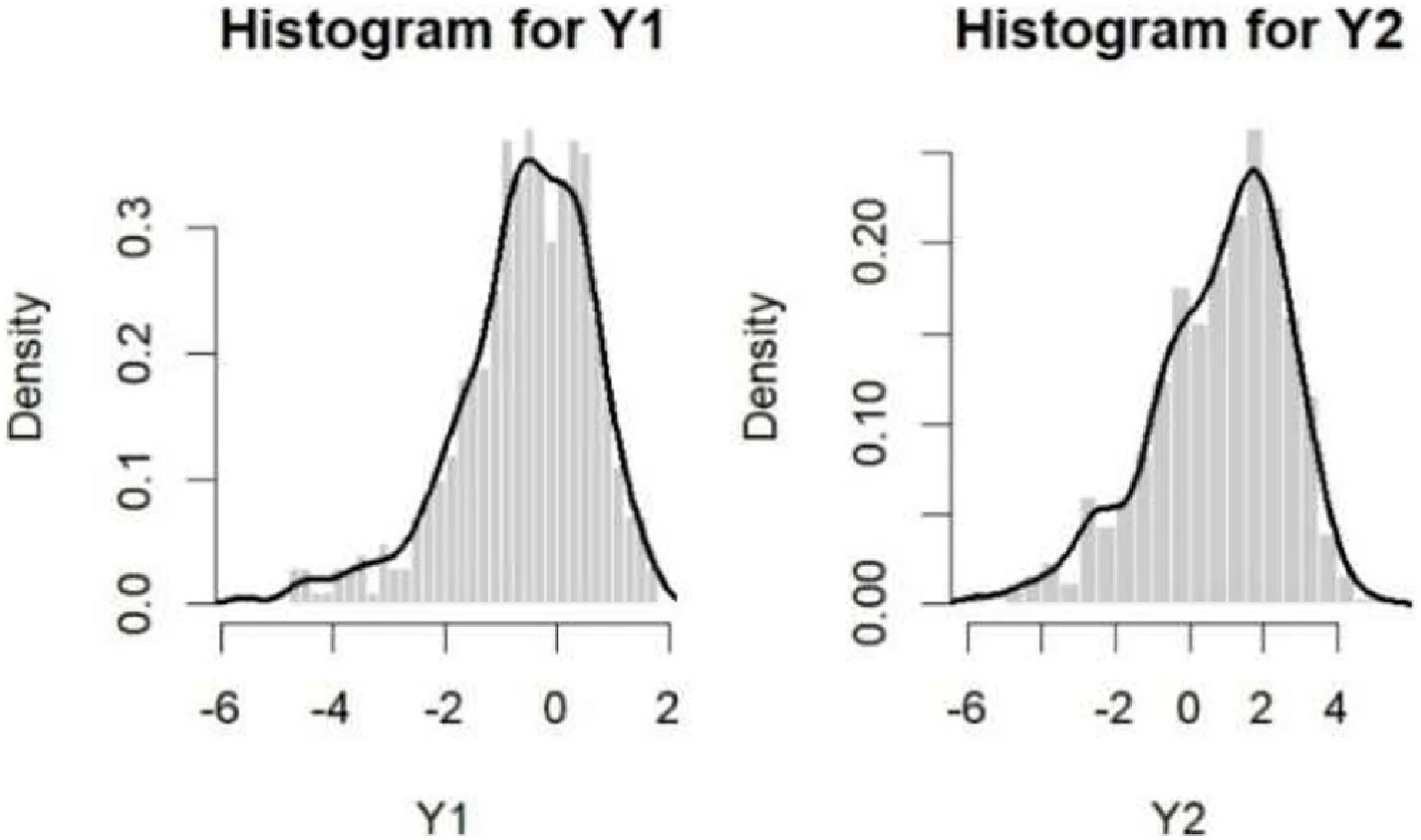
Histograms of the simulated $Y_{1}$ and $Y_{2}$ Observation.

Based on Fig. 3 on the histogram $Y_{1}$, most of the data is concentrated around values between -1 to 1, with the peak density being close to the value of 1. However, the distribution appears to have a longer tail to the left, which is up to about -6. This indicates that $Y_{1}$ does not spread symmetrically like normal distributions, but has the characteristic of pressing to the left or has a longer left tail. This condition corresponds to the characteristic of Generalized Log-Gamma at =1, which is capable of generating continuous data with an asymmetrical shape.

Histogram $Y_{2}$ indicates that the center of the distribution shifts to the right compared to $Y_{1}$. Most values of, $Y_{2}$ are around 0 to 3, with a peak density of around a value of 2. As a result, the value of $Y_{2}\;$tends to be greater than $Y_{1}$. In other words, the parameter $\mu_{2}=2\;$causes the distribution $Y_{2}$ to shift to the right. Distribution $Y_{2}$ also appears asymmetrical, with a fairly long left tail up to about -6. This shows that although $Y_{2}$ shifted to the right, the skewness characteristic of the Generalized Log-Gamma distribution remains. The results of the simulation correspond to the parameters used. The parameter $\mu_{1}=0\;$causes the distribution $Y_{1}\;$to be centered around 0. The parameter $\mu_{2}=2$causes the distribution $Y_{2}$ to shift to the right, so that the center of the distribution is around 2. The parameters $\sigma_{1}=1$ and $\sigma_{2}=1\;$make the data spread at medium scale. Meanwhile, the λ=1 parameter produces an asymmetrical form of distribution, with a longer tail to the left.

Based on Fig. 4 the correlation value displayed *r = 0.6868*, this means that there is a strong positive relationship between *Y_1_* and *Y_2_* When the value of *Y_1_* increases, the value of *Y_2_* tends to increase. This can also be seen from the regression line that goes uphill from the bottom left to the top right. Thus, *Y_2_* is not completely free from *Y_1_*, as *Y_1_* goes directly into the formation of *Y_2_*. This is what causes the two variables to have a positive relationship. The point spread does not form a truly symmetrical pattern. The dots are seen to be more concentrated in the range *Y_1_* around -1.5 to 1, and *Y2* around -1 to 3. However, there are some extreme points, especially at very small *Y_1_* values and low *Y_2_*, as well as some points with very low *Y_2_* even though *Y_1_* is around zero. This shows that the simulation data still has the characteristics of skewness and a fairly long tail, according to the properties of the Generalized Log-Gamma distribution.

**Fig. 4:**
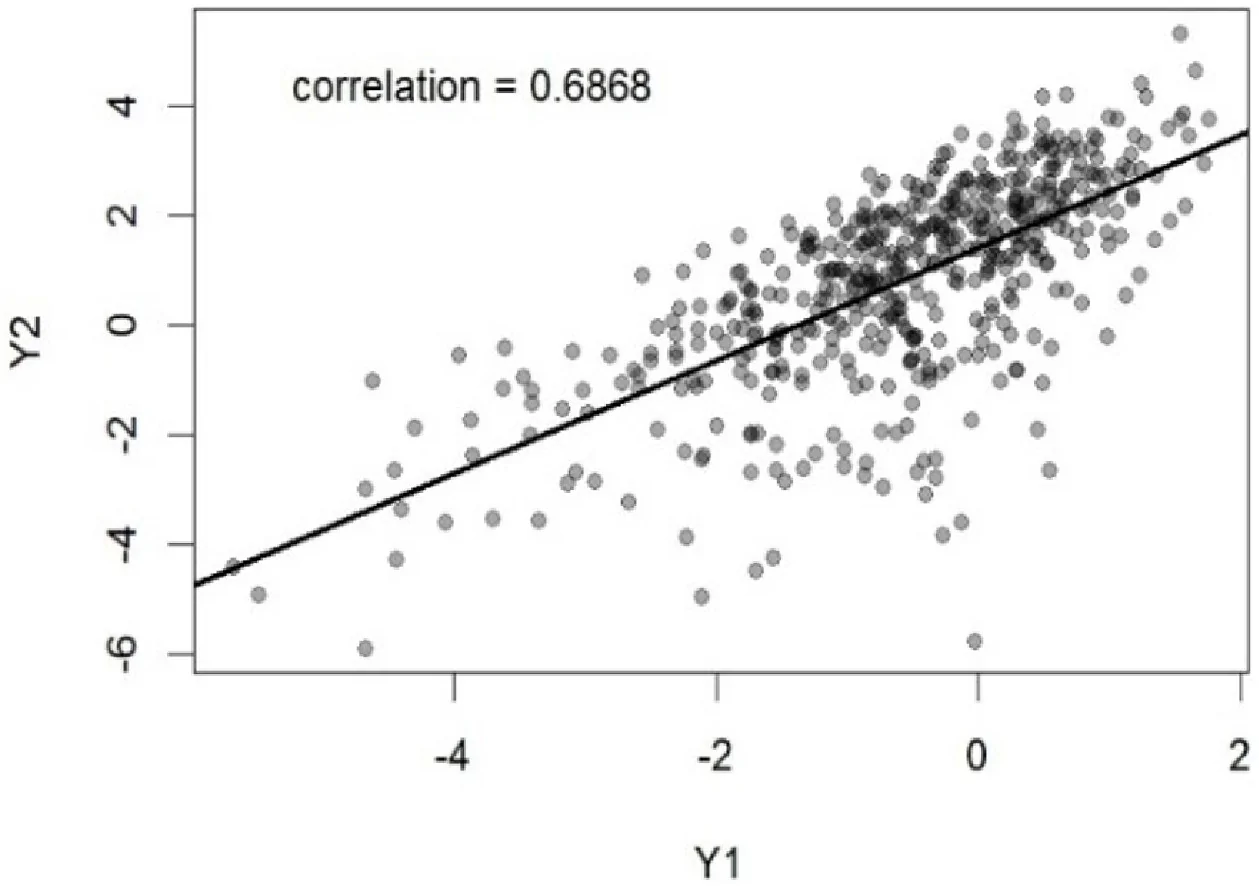
Scatter plot of the simulated BGLG observations.

The final stage provides an illustrative validation of BGLG distribution under a single simulation setting.

The data used in this validation were not obtained from an external empirical source but were generated through a simulation based on the proposed BGLG model. A total of 500 paired observations, $\left(Y_{1i},Y_{2i}\right)$, were generated using the parameter settings $\mu_{1}=0$, $\mu_{2}=2$, $\sigma_{1}=\sigma_{2}=1$, and a common shape parameter λ=1. The dependence between the two responses was introduced through the BGLG construction, in which $Y_{2}$was formed in relation to $Y_{1}$. Consequently, the generated data retained asymmetric marginal distributions while exhibiting a positive association between the two variables. These results provide evidence for the specified simulation setting and do not represent a comprehensive evaluation of the method.

In this procedure generally following step 5, the simulated observations were transformed sequentially into Gamma variables, uniform probabilities, and standard normal scores, and the resulting squared distances were compared with the theoretical quantiles of a chi-square distribution with two degrees of freedom. These validation stages were used to determine whether the proposed construction produced a valid joint density, preserved the intended asymmetric marginal behavior, represented positive dependence, and adequately described data generated from the BGLG model.

Fig. 5 show the chi-square plot based on the previously generated Y_1_ and Y_2_ data. The chi-square plot in Fig. 5 of the BGLG shows the observation points following the reference line quite closely. This indicates that the square spacing of the BGLG transformation results is close to the distribution $\chi_{2}^{2}$. Small deviations are visible in the upper part of the tail, but are not dominant. The plotted points follow the reference line reasonably closely, with minor deviations in the upper tail. This provides graphical support for the adequacy of the BGLG distribution under the evaluated simulation setting. However, the chi-square plot should be interpreted as a graphical diagnostic rather than as a formal goodness-of-fit test. *.*

**Fig. 5:**
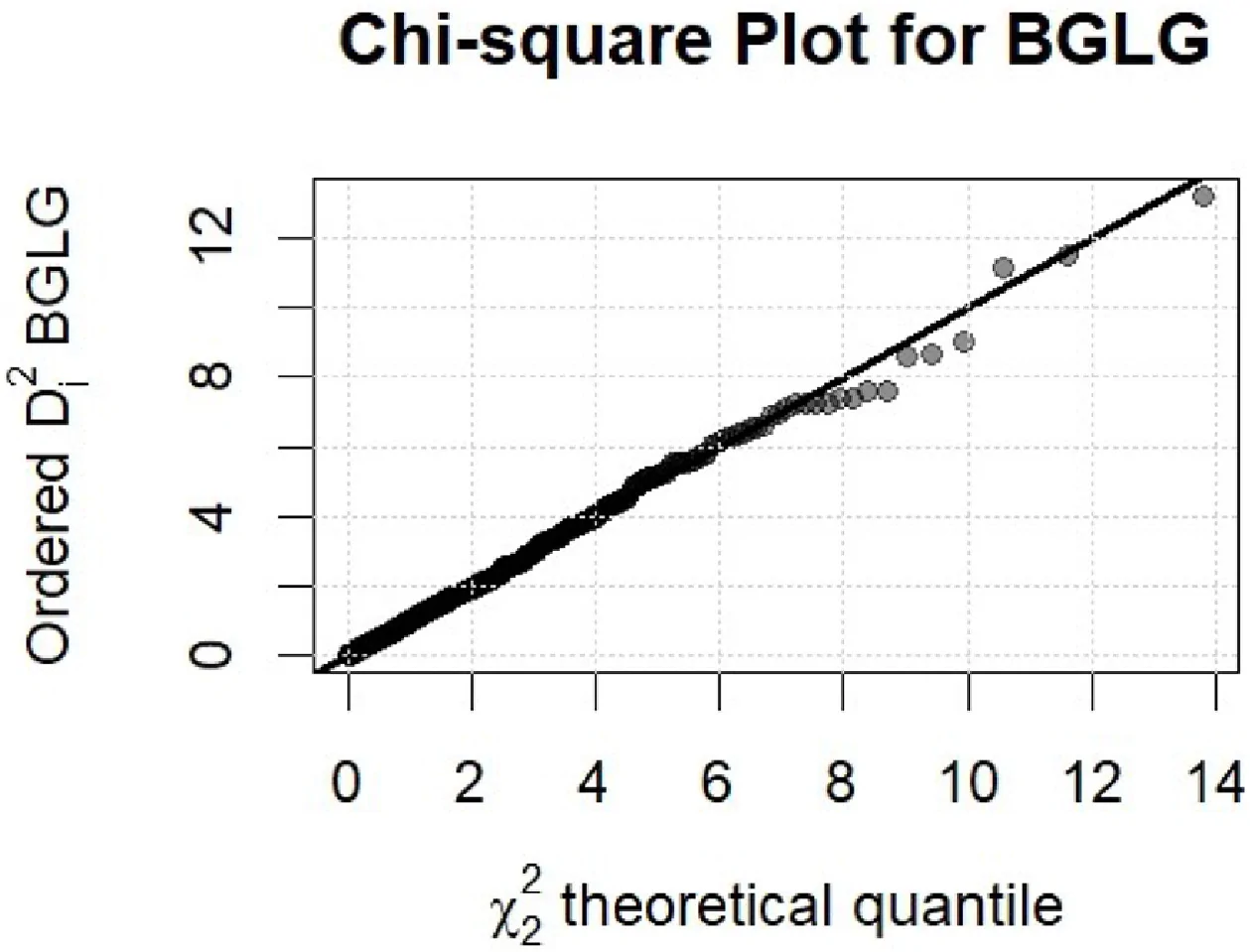
Chi-square diagnostic plot for the simulated BGLG observations.

### Limitations

The proposed method is currently limited by the assumption of a common shape parameter for both marginal distributions and by validation under a single simulation setting. The chi-square diagnostic provides graphical evidence of model adequacy but does not yield a formal test statistic or *p*-value. In addition, BHHH estimation may be sensitive to initial values and near-singularity of the outer-product-of-gradient matrix. The method has not yet been compared with competing bivariate distributions or applied to empirical data. Future work should examine broader simulation scenarios, formal goodness-of-fit tests, alternative optimization methods, real-data applications and apply together with the regression method.

## Ethics statements

This manuscript represents the authors’ original work and has not been published previously, in whole or in part, nor is it currently under consideration for publication by another journal. All authors have read and approved the final manuscript and have agreed to its submission to MethodsX. The authors declare that they have no competing financial interests, personal relationships, professional affiliations, or conflicts among themselves that could have influenced the conduct, interpretation, or reporting of this research.

## Related research article

***For a published article:***

## CRediT authorship contribution statement

**Arief Rachman Hakim:** Conceptualization, Methodology, Writing – original draft, Software. **Purhadi:** Conceptualization, Methodology, Supervision, Validation. **Jerry Dwi Trijoyo Purnomo:** Supervision, Writing – review & editing, Validation.

## Declaration of competing interest

The authors declare that they have no known competing financial interests or personal relationships that could have appeared to influence the work reported in this paper.

## Data Availability

Data will be made available on request.

## Appendix Supplementary materials

Supplementary material *and/or* additional information [OPTIONAL] Supplementary Material S1 BGLG: Complete R code used to generate the simulated data and conduct the simulation analyses, including BHHH parameter estimation and the transformation-based chi-square diagnostic.
